# Concentration of DNA at the Cell Surface Dictates Transfection Efficacy: A Hyperbranched Poly(β-Amino Ester) Mediated Strategy for Enhanced Lentivirus Production

**DOI:** 10.3390/polym18091015

**Published:** 2026-04-22

**Authors:** Miao Wei, Liang Yao, Xingyue Wang, Meilin Guo, Haonan Li, Guang Chen, Xianqing Wang, Xi Wang, Wenxin Wang, Zhonglei He

**Affiliations:** 1 School of Medicine, Anhui University of Science and Technology, Huainan 232001, China; miaow17@126.com (M.W.); 13696515514@163.com (X.W.); 2 Institute of Precision Medicine (AUST-IPM), Anhui University of Science and Technology, Huainan 232001, China; 2023169@aust.edu.cn (X.W.); wenxin.wang@ucd.ie (W.W.); 3 Charles Institute of Dermatology, University College Dublin, D04 V1W8 Dublin, Ireland; liang.yao@ucdconnect.ie (L.Y.); xianqing.wang@ucd.ie (X.W.); 4 ST PHI Therapeutics Co., Ltd., Hangzhou 310051, China; meilin.guo@stphibio.com; 5 ACROBiosystems Inc., Beijing 100176, China; haonan.li@acrobiosystems.com; 6 Zhejiang Key Laboratory of New Drug Development for Central Nervous System Diseases, School of Medicine, Taizhou University, Taizhou 318000, China; gchen@tzc.edu.cn; 7 School of Public Health, Anhui University of Science and Technology, Huainan 232001, China

**Keywords:** hyperbranched poly(β-amino ester), gene delivery, plasmid DNA, lentivirus, transient transfection

## Abstract

Hyperbranched poly(β-amino ester) (HPAE) is identified as a unique non-viral carrier capable of sustaining high-efficiency transfection under elevated plasmid concentrations, overcoming the aggregation and toxicity limitations of conventional lipid and PEI reagents. We demonstrate that transfection enhancement is driven by concentration-dependent synergism between membrane accumulation and endosomal escape. Guided by this mechanism, a half-volume transfection strategy was established to transiently elevate plasmid concentration without compromising cell viability, enabling superior lentivirus yield and purity. These findings define plasmid concentration as a previously overlooked regulatory axis in nanoparticle-mediated gene delivery and position HPAE as a high-performance platform for scalable therapeutic vector production.

## 1. Introduction

Gene therapy has established significant potential in treating genetic disorders, cancers, and infectious diseases [1,2]. Its success largely depends on the efficiency and safety of the gene delivery system [3,4,5,6]. Viral vectors remain effective yet are limited by immunogenicity and insertional mutagenesis risks, while lipid-based reagents often suffer from suboptimal efficiency and cytotoxicity. Therefore, the development of safe and efficient non-viral delivery systems remains a central challenge in advancing gene therapy [7,8,9].

In non-viral gene delivery systems, plasmid DNA (pDNA), as a commonly used nucleic acid therapeutic agent in gene therapy, plays a crucial role in the success or failure of treatment [10]. pDNA is one of the most widely used nucleic-acid payloads, and its delivery efficiency is not only determined by vector properties but is strongly dependent on the plasmid concentration at the cell surface [11,12,13]. Higher concentration promotes membrane adsorption, enhances cellular uptake, and ultimately increases transfection efficiency. However, conventional transfection strategies often fail to regulate this parameter effectively, leading to limited efficiency even at increased total DNA doses. Among numerous non-viral vectors, hyperbranched poly(β-amino ester) (HPAE) has become a highly promising delivery vector due to its regulable branched structure, cationic properties and inherent biodegradability [14,15]. Their abundant amine groups enable strong electrostatic interaction with pDNA, promoting membrane enrichment and facilitating endosomal escape [16,17,18]. In this study, HPAE was selected as the research platform based on our previous systematic work. We have successfully developed and optimized a series of HPAE materials and conducted a comprehensive investigation and optimization of their branching unit distribution, charge density, and gene delivery performance. Previous studies have shown that such materials possess excellent comprehensive properties suitable for nucleic acid delivery, including structural designability, good safety, and high delivery efficiency, which are crucial for investigating the concentration-dependent delivery behavior [14,19]. Nevertheless, to date, no systematic study has elucidated the role and pattern of plasmid concentration-dependent mechanisms within HPAE-mediated delivery systems, provided in-depth validation of the enhanced transfection mechanisms, or explored its application in improving lentivirus production.

Lentivirus (LVs) are an attractive gene delivery vehicle for a wide range of applications, and transient transfection efficiency directly governs vector yield [20,21,22,23,24]. However, no study has clarified whether enhancing plasmid concentration at the cell surface through HPAE delivery could improve LV production. To address this gap, the present work investigates concentration-dependent transfection using HPAE and shows its translational potential in lentivirus manufacturing.

**Scheme 1:**
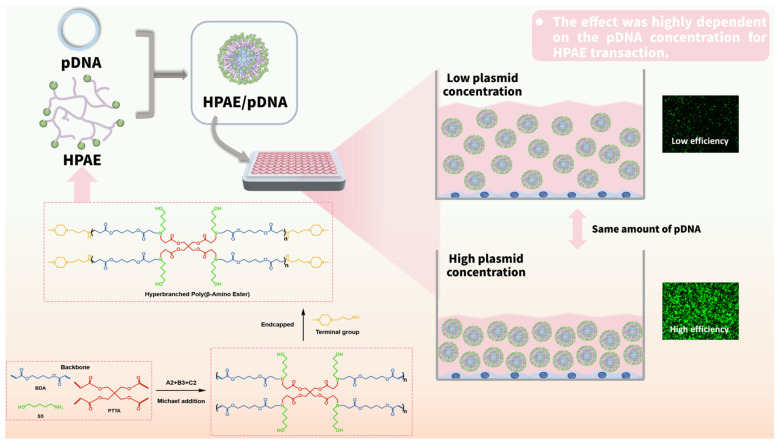
Synthesis of Hyperbranched Poly(β-amino ester) and Its Plasmid Concentration-Dependent Transfection Mechanism.

In this study, we propose and systematically validate, for the first time, the concentration-dependent mechanism of plasmid delivery mediated by HPAE (Scheme 1). Through physicochemical characterization, we revealed the unique properties of HPAE and explained the physical basis for its highly efficient delivery even under high-concentration conditions. Cellular-level experiments further elucidated the essential mechanisms involved. Moreover, we applied this principle to lentivirus production and developed a novel strategy that achieved dual optimization of both yield and purity. This work provides validation at the fundamental mechanistic level and offers a feasible new approach for scalable lentivirus production in practical applications.

## 2. Materials and Methods

### 2.1. Polyplex Size

According to the previous research of our team, the four-armed HPAE synthesized by the team earlier (Scheme 1) was used for subsequent experiments. To measure the size of polyplexes via dynamic light scattering (DLS), the polymer was first dissolved in dimethyl sulfoxide (DMSO) to prepare a stock solution (100 mg/mL). This was then diluted with 25 mM sodium acetate buffer to prepare a working solution at a concentration of 2 mg/mL. DNA was diluted with the same buffer to a concentration of 0.1 mg/mL. Equal volumes of the polymer and DNA solutions were thoroughly mixed to form polyplexes. For commercial transfection reagents (Lipofectamine 3000 from Thermo Scientific, Waltham, MA, USA and jetPEI from Polyplus, Strasbourg, France), polyplexes were prepared strictly following the manufacturers’ protocols. The particle size of the resulting polyplexes was measured using a Zetasizer Pro (Malvern Panalytical, Malvern, UK). For DLS measurements, 100 μL of the sample solution was loaded into a quartz cuvette with a 10 mm path length, and the intensity of scattered light was recorded at a detection angle of 173°. To evaluate colloidal stability, the polyplexes were incubated at 37 °C, and their particle size and polydispersity index (PDI) were monitored at time intervals of 0, 30, 60, 90, and 120 min. All experiments were performed in triplicate.

### 2.2. Polyplex Sedimentation Kinetics

A quantitative analytical method based on heparin sodium salt cleavage was employed to evaluate the sedimentation kinetics of the polymeric complex. Heparin is a highly negatively charged sulfated linear glycosaminoglycan, whose strong negative charge primarily stems from the abundance of sulfate groups in its structure. When heparin comes into contact with cationic polymer–DNA nanoparticles, it can competitively bind to the cationic polymer via electrostatic interactions, thereby disrupting or even destroying the stable complex originally formed between the cationic polymer and the negatively charged DNA [25]. A heparin dosage and incubation time assay was first performed to determine the optimal heparin conditions for each polymer complex. HPAE, Lipofectamine 3000, and jetPEI polyplexes were prepared in physiological buffer at a fixed polymer/DNA mass ratio (total volume 100 μL). The final concentration of heparin was 200 μg/mL. Heparin was added at three different mass ratios relative to DNA (80:1, 100:1, and 120:1), and samples were incubated at 37 °C for 1 h or 2 h. From each group, 90 μL of sample was collected and mixed with 90 μL of PicoGreen dye (200 × diluted in 1 × TE buffer), followed by incubation at room temperature in the dark for 15 min. A 0% release control (buffer and medium only) and a 100% release control (buffer and medium with an equivalent mass of free DNA) were included. Fluorescence intensity was measured using a microplate reader at an excitation wavelength of 485 nm and an emission wavelength of 535 nm. Based on the optimization results, the best heparin ratio and dissociation time were applied in the formal sedimentation experiment. Polyplexes were prepared at the same ratio (total volume 100 μL) and incubated at 37 °C. At predetermined time points (0, 1, 2, 3, 4, and 5 h), 50 μL of supernatant was carefully withdrawn from each sample and transferred to a black 96-well plate. Heparin solution was then added to the collected supernatant at the optimized ratio, followed by incubation for the optimized dissociation time to ensure complete polyplex dissociation and DNA release. Subsequently, 50 μL of PicoGreen dye was added to each well, and samples were incubated at room temperature in the dark for 15 min before fluorescence measurement at 485/535 nm.

The sedimentation rate was calculated using the following formula:

$$\mathrm{Sedimentation}\;\mathrm{rate}\;(\%)=\left[1-\frac{F_{t}}{F_{0}}\right]\times 100\%$$

where *F_t_* represents the fluorescence intensity at time t, and *F*_0_ represents the fluorescence intensity at 0 h. All experiments were performed in triplicate, and the results are presented as mean ± SEM.

### 2.3. Polyplex Stability

To validate the stability of the polymer HPAE, HEK293T cells were seeded in a 96-well plate at a density of 2.5 × 10^4^ cells per well, with each well containing 100 μL of culture medium, and cultured until they reached 70–80% confluency. Polyplexes were prepared at three mass ratios—15:1, 20:1, and 25:1 (*w*/*w*)—using the mixing method described above. After incubation at 37 °C for 0, 30, 60, 90, and 120 min, the culture medium was rapidly removed from the wells, and the polyplex solutions were added. The cells were then cultured for 48 h. GFP fluorescence intensity and cell status were observed using a high-content fluorescence microscope (Thermo EVOS M7000, Waltham, MA, USA). Following image acquisition, the GFP fluorescence was analyzed and quantified using ImageJ (fiji-win64).

### 2.4. In Vitro Transfection

The culture medium was DMEM supplemented with 10% Fetal Bovine Serum (FBS) and 1% Penicillin-Streptomycin (PS). The cell lines used included HEK293T (ATCC), U251-MG, A549, 3T3, HeLa, LN229, and MDA-MB-231 (National Collection of Authenticated Cell Cultures). For transfection, cells were seeded in a 96-well plate at a density of 2.5 × 10^4^ cells per well in 100 μL of medium one day in advance and cultured until they reached 70–80% confluency. Transfection was performed the following day.

#### 2.4.1. Transfection Efficiency at Different Plasmid Concentrations

eGFP-DNA (1 mg/mL, GENERAL BIOL, Chuzhou, China) was used for transfection. For the medium-change groups, with each well containing 0.2 μg, 0.3 μg, 0.4 μg, or 0.5 μg of DNA diluted in NaOAc buffer (final concentration 25 mM). According to a weight ratio of 20:1 (*w*/*w*), the polymer was also diluted in NaOAc buffer. The polymer solution was then added to the DNA solution at a 1:1 volume ratio, mixed gently by pipetting, and incubated for 5 min to allow polyplex formation. The polyplexes were subsequently added into DMEM medium to reach final volumes of 40 μL, 60 μL, 80 μL, or 100 μL, the plasmid concentration was fixed at 5 μg/mL. For the control groups (constant medium volume), the culture medium volume was maintained at 100 μL, the DNA amount per well was the same as he medium-change groups, while the DNA concentration was varied to 2 μg/mL, 3 μg/mL, 4 μg/mL, or 5 μg/mL. The exact mass of DNA was diluted in sodium acetate buffer, polyplexes were prepared as described above, and then diluted with DMEM medium to a final volume of 100 μL. In total, 8 groups were tested, with six replicate wells for the medium-change groups. After 2 h of transfection, the medium-change groups were replenished with fresh DMEM to a final volume of 100 μL, and continued transfection was maintained for an additional 46 h. At the end of transfection, GFP expression and cell morphology were observed and imaged using a high-content fluorescence microscope (M7000). GFP fluorescence intensity was quantified using ImageJ software.

Two conditions were established for the high plasmid dose experiments: a medium-change group (increasing concentration through medium adjustment) and a plasmid-dose group (increasing concentration by adjusting the mass of DNA) as a control. All three medium change groups used 0.5 μg of DNA per well, with medium volumes of 100 μL, 50 μL, and 25 μL, respectively, corresponding to concentrations of 5 μg/mL, 10 μg/mL, and 20 μg/mL. The control groups were tested with three plasmid loading amounts: 0.5 μg, 1 μg, and 2 μg per well, corresponding to concentrations of 5 μg/mL, 10 μg/mL, and 20 μg/mL, respectively. Three replicate wells were set up for each condition. The preparation method of the polyplexes was as described previously. Each condition was performed in triplicate wells. Polyplex preparation was conducted as described above. After two hours of transfection, the medium-change groups were supplemented with fresh medium to a final volume of 100 μL, and continued transfection was maintained for an additional 46 h. At the end of transfection, cells were imaged, and GFP expression was quantified using ImageJ.

For the control groups, Lipofectamine 3000 and jetPEI were used according to the manufacturers’ recommended protocols without further re-optimization. These conditions are widely accepted as standard and are intended to ensure reproducibility and comparability across studies. Transfection was performed following the same experimental design, and after 46 h, GFP expression was observed by fluorescence microscopy and quantified using ImageJ.

To further validate the efficiency of gene delivery, this study also conducted transfection experiments using a plasmid encoding firefly luciferase as a reporter gene. This experiment was used to quantitatively assess the expression levels of the exogenous gene following transfection. The transfection method for the luciferase plasmid was the same as that used in the aforementioned eGFP transfection experiment. After transfection, luciferase expression was quantified using the Firefly Luciferase Reporter Gene Assay Kit (Beyotime, RG005, Shanghai, China) following the manufacturer’s protocol. Briefly, 100 μL of lysis buffer was added to each well, and after 15 min of incubation, 100 μL of fully lysed cell suspension was collected and centrifuged at 10,000× *g* for 5 min. The supernatant was mixed with the detection reagent, and luminescence intensity was measured using a multimode microplate reader equipped with a luminometer.

To further evaluate the transfection efficiency and quantitatively determine the proportion of successfully transfected cells, this study also employed eGFP-DNA as a reporter gene for transfection. The experimental groups and transfection procedures were completely consistent with those described in the aforementioned transfection experiments. Forty-eight hours after transfection, the cells were harvested by trypsin (Biosharp, Beijing, China) digestion and resuspended in ice-cold phosphate-buffered saline (PBS) (MeilunBio, Dalian, China). The proportion of eGFP-positive cells and median fluorescence intensity (MFI) were analyzed using a flow cytometer (CytoFLEX SRT, Beckman Coulter, Brea, CA, USA). Ten thousand cells were collected per sample, and data analysis was performed using GraphPad (11.0.1 version).

#### 2.4.2. Cell Viability

Cell viability under various conditions was assessed using the AlamarBlue assay (Thermo Scientific, cat. #DAL1025, Waltham, MA, USA) according to the manufacturer’s protocol. Briefly, 10 µL of the reagent was added to each well containing transfected cells, followed by incubation at 37 °C until the color changed from blue to pink (approximately 1 h). Absorbance was measured at 530/595 nm using a multi-mode microplate reader. For data analysis, three wells from the medium-change-only group were treated with the reagent to represent 100% viability, while three additional wells without the reagent served as the 0% reference. Cell viability was evaluated by comparing the results against those of these controls.

#### 2.4.3. Polyplex Cellular Uptake and Endosomal Escape Efficiency

According to the manufacturer’s instructions, eGFP-DNA was labeled with Cy3, a red fluorescent dye, using a commercial labeling kit (Label IT Nucleic Acid Labeling Technology, QIHE Medical, Suzhou, China). The final concentration of Cy3-DNA was 0.5 mg/mL. 2.5 × 10^4^ HEK293T cells were seeded per well in a 96-well plate for cellular uptake experiments. Gene transfection was performed using the HPAE/pDNA system at a ratio of 20:1 (*w*/*w*). After 1.5 h of transfection, the culture medium was removed, and live-cell nuclear staining was performed using NucBlue™ Live Cell Stain (Thermo Scientific, Waltham, MA, USA). Two drops of reagent were added per 1 mL of medium, and 100 μL was added to each well. After 20 min of incubation, multiple regions per well were imaged at high magnification to observe red (Cy3-DNA) and blue (nuclei) fluorescence for evaluation of Cy3-DNA uptake.

To assess the endosomal escape capacity of HPAE/pDNA polyplexes at different plasmid concentrations, a HEK293T cell line stably expressing Galectin-8 fused to Yellow Fluorescent Protein (Gal8-YFP) was used [26]. This reporter system allows visualization of endosomal membrane damage, as Gal8 rapidly accumulates at disrupted endosomes to form fluorescent puncta, serving as a sensitive indicator of endosomal membrane disruption. Cells were seeded at 3 × 10^4^ cells per well in a 96-well plate and cultured at 37 °C with 5% CO_2_ for 24 h until reaching 70–80% confluency. HPAE/pDNA polyplexes were applied under three transfection conditions and incubated with the cells for 4 h: (i) 0.5 μg DNA, 5 μg/mL; (ii) 0.2 μg DNA, 5 μg/mL; and (iii) 0.2 μg DNA, 2 μg/mL. Endosomal membrane disruption events were monitored by confocal microscopy at 40× magnification (Olympus IX81 microscope, Tokyo, Japan), with six fields of view analyzed per well.

Image analysis was performed using ImageJ software. Fluorescent spots were identified automatically by setting a threshold of puncta intensity, and the number of cells per field was simultaneously counted. Endosomal membrane disruption was expressed as the average Gal8 spots intensity per cell per field, calculated as:

$$\mathrm{Avg}.\;\mathrm{Gal}8\;\mathrm{spots}\;\mathrm{level}=\frac{\mathrm{Total}\;\mathrm{spots}\;\mathrm{fluorescence}}{\mathrm{Total}\;\mathrm{cell}\;\mathrm{count}}$$

Data are presented as mean ± SEM, and statistical significance was assessed by one-way ANOVA.

### 2.5. Lentivirus Production Using HPAE-Based New Transfection Strategies

HEK293T cells were transfected with HPAE using a four-plasmid lentivirus packaging system. The transfer plasmid LV010 was used, and cells were maintained in adherent culture at a seeding density of 5.5 × 10^4^ cells/cm^2^ in 2% FBS–Opti-MEM medium. Three commercial transfection reagents, LVMAX (Thermo Scientific, Waltham, MA, USA), PEIpro (Polyplus, Strasbourg, France) and BrPERfect for Virus (Branca Bunús Ltd., Dublin, Ireland), were included as controls. The specific conditions and procedures for the control reagents shall be carried out in accordance with their respective instructions.

Cells were passaged when confluence exceeded 80% and seeded into T75 flasks (7.5 mL medium). After 48 h, lentivirus packaging was performed using a plasmid/HPAE ratio of 5:1. 6 h post-transfection, the culture medium was supplemented to a final volume of 15 mL. Each condition was tested in triplicate. For the control groups, cells were seeded in T75 flasks (15 mL medium) and, after 48 h, were transfected with each of the three reagents. At 6 h post-transfection, the medium was replaced with fresh medium. Each reagent group included three replicates.

Collect the cell culture supernatant 24 h after adding or replacing the fresh culture medium for analysis of the biological titer, p24 physical titre, and impurity levels of host cell DNA (HCD) and host cell protein (HCP).

### 2.6. Statistical Analysis

Curve plotting and statistical analysis were performed using Prism 9 (GraphPad). Statistical analysis was conducted using an ordinary one-way and two-way analysis of variance (ANOVA), followed by Tukey’s multiple comparisons test. All the data were presented as mean ± standard error of the mean (s.e.m.), and a *p* value below 0.05 was considered statistically significant.

## 3. Result and Discussion

### 3.1. Polyplex Characterization

**Figure 1:**
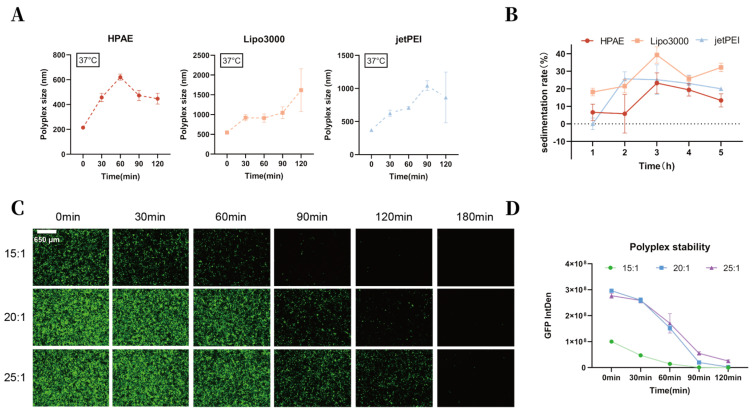
Characterization of the physicochemical properties of HPAE, Lipofectamine 3000, and jetPEI. (**A**) Size stability of HPAE, Lipo3000, jetPEI polyplexes over 120 min in physiological buffer at 37 °C (*n* = 3 biological independent replicates). (**B**) Sedimentation kinetics of three polyplexes within 5 h (*n* = 3 biological independent replicates). (**C**,**D**) Stability of polyplex in physiological environments (**C**) Fluorescence images of Polyplex transfected with HEK293T at 37 °C for different periods of time (*n* = 3 biological independent replicates). Scale bars, 650 μm. (**D**) Quantification of eGFP expression in (**C**) using ImageJ software (*n* = 3 biological independent replicates).

To clarify the delivery performance advantages of HPAE, we first systematically characterized its physicochemical properties and conducted a comparative analysis with commercial transfection reagents, Lipofectamine 3000 and jetPEI. Our group has previously reported the synthesis, structural optimization, and physicochemical characterization of HPAEs, establishing a multifunctional polymer library encompassing diverse branched architectures for gene delivery applications [27,28,29]. Following these studies, we selected a representative HPAE as shown in Scheme 1 to investigate its unique features and underlying mechanisms in adherent cell transfection. Previous research by Dezhong et al. [15] indicated that the optimal formulation for highly efficient gene transfection with HPAEs typically involves a polymer/DNA weight ratio of 20:1 (*w*/*w*). Therefore, we initially used a 20:1 (*w*/*w*) ratio to evaluate the gene transfection efficiency of various HPAEs. In contrast, commercial transfection reagents were applied according to their optimized protocols. Dynamic light scattering (DLS) analysis showed that HPAE/pDNA polyplexes maintain high colloidal stability at physiological temperature (37 °C), exhibiting time-dependent size evolution within the nanoscale range (Figure 1A). Although moderate aggregation was observed during incubation, the polyplexes did not undergo irreversible growth or precipitation, and their size partially decreased over time, remaining stably dispersed at the nanoscale. In stark contrast, Lipofectamine 3000 and jetPEI complexes underwent irreversible aggregation, rapidly exceeding micrometer-scale sizes. HAPE result is consistent with our group’s previous work on branching-unit distribution (BUD), which proved that optimized BUD improves complex stability, charge buffering, and colloidal uniformity. This stability underpins its efficient, concentration-dependent transfection [14,30,31]. In contrast, due to inherent structural limitations, Lipofectamine 3000 and jetPEI fail to achieve comparable performance [32].

To systematically evaluate the suspension stability of HPAE and its role in the enrichment of plasmids on the cell surface, we compared the kinetic behaviors of HPAE with two commercial transfection reagents (Lipofectamine 3000 and jetPEI) over 5 h by measuring the sedimentation rate of polyplexes. In this experiment, a method based on heparin sodium to lyse polymers for quantifying the polypolymers in the samples was used to assess the sedimentation stability of the complexes. To establish the optimal conditions for heparin-induced DNA dissociation in the sedimentation assay, we first screened and optimized the heparin ratio and incubation time for three polymers (Figure S1). Based on these results, subsequent sedimentation experiments used a heparin/DNA ratio of 120:1 with dissociation times of 2 h for HPAE and Lipofectamine 3000, and 1 h for jetPEI, to ensure complete polyplex dissociation and accurate sedimentation DNA quantification. Sedimentation analysis (Figure 1B) revealed pronounced differences among the delivery systems. HPAE exhibited significantly delayed sedimentation, with an average sedimentation rate of 6.56% at 1 h, markedly lower than that of Lipofectamine 3000 (16.93%). This low sedimentation profile remained stable up to 5 h. Although jetPEI displayed a relatively smooth overall curve, it exhibited a rapid increase in sedimentation rate during the initial 2 h, reaching approximately 25%, indicative of pronounced early-stage sedimentation. This may reduce cell contact opportunities and impair transfection efficiency [33]. In contrast, Lipofectamine 3000 consistently showed high sedimentation rates throughout the observation period, accompanied by substantial intra-group variability, reflecting instability in its polyplex suspension behavior. Lipofectamine3000 tends to undergo rapid fusion and irreversible aggregation due to lipid–lipid coalescence [34], whereas jetPEI shows continuous size growth driven by uncontrolled polymer–polymer bridging and low hydration compatibility [35], both ultimately accelerating sedimentation and reducing effective cell engagement. This sustained high sedimentation tendency likely leads to rapid precipitation, shortening the effective contact time with cells and consequently compromising transfection efficiency [36,37]. HPAE showed superior low and stable sedimentation properties, with minimal fluctuation throughout the range of observation, indicating better reproducibility and system stability.

To further assess the structural integrity and stability of HPAE under physiological conditions, we examined the stability of HPAE/pDNA polyplexes prepared at different polymer-to-DNA mass ratios (15:1, 20:1, and 25:1, *w*/*w*), incubated at 37 °C. Polyplexes containing 0.5 μg of eGFP-encoding plasmid were incubated in DMEM medium for 0 to 120 min and then transfected into HEK293T cells. Analysis at 48 h post-transfection showed that the stability of the polyplexes was significantly related to the polymer-to-DNA mass ratio. Notably, the 20:1 and 25:1 ratio showed prolonged structural stability exceeding 90 min during incubation (Figure 1C,D). Consistent with prior in vitro and in vivo studies showing the high transfection efficiency of HPAE [15], these stability results further support that the 20:1 ratio provides an optimal balance between structural robustness and functional delivery efficiency. Based on these findings, all subsequent mechanistic and application-focused experiments used this standardized formulation to ensure reproducibility and translational relevance.

### 3.2. Plasmid Concentration Affects Transfection Efficiency

**Figure 2:**
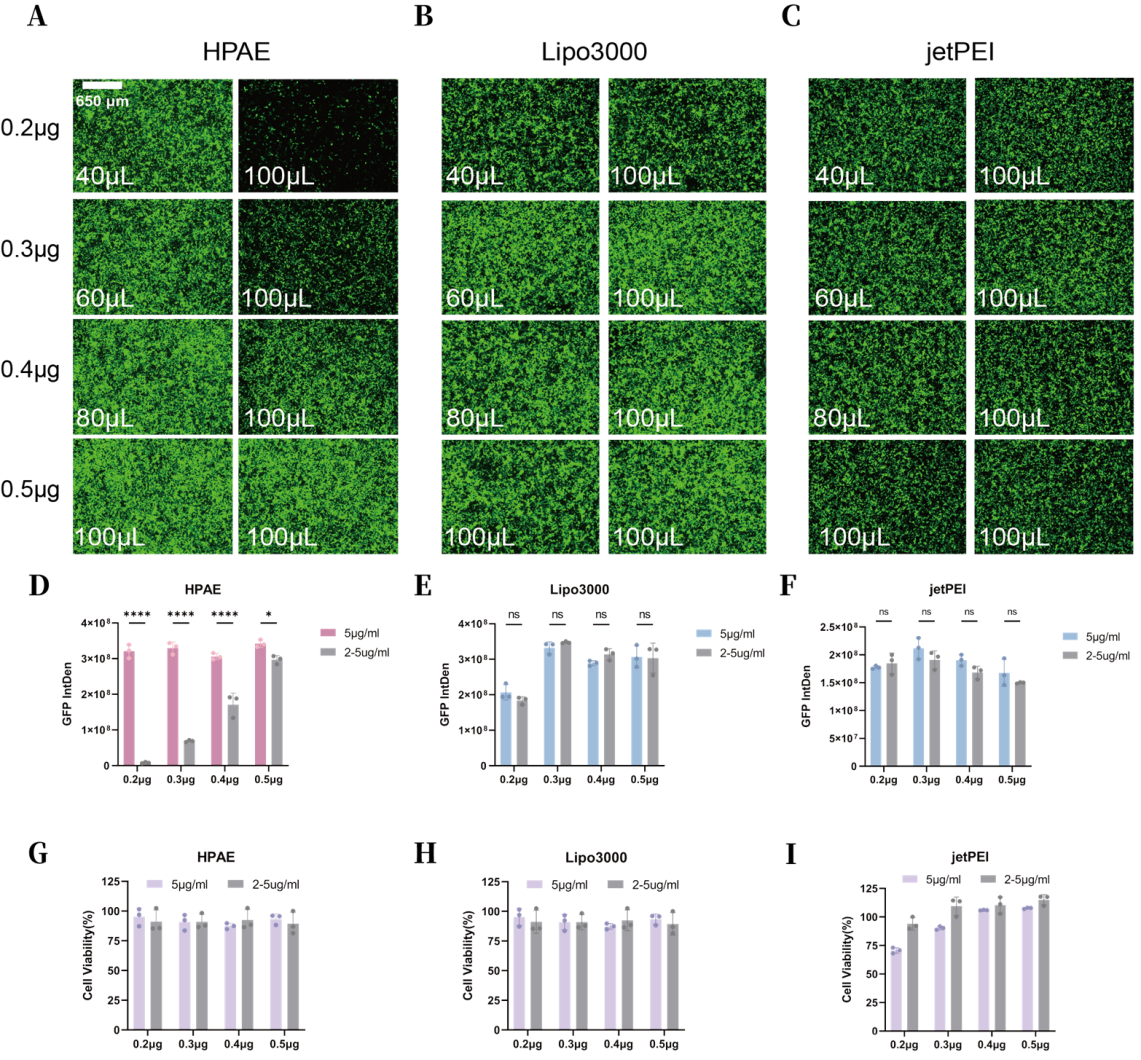
Transfection performance under plasmid mass reduction and concentration maintenance strategies. (**A**) Fluorescence images of HEK293T cells transfected with HPAE/pDNA polyplexes. Left panel: plasmid mass gradient (0.2–0.5 μg/well) with increasing medium volume (40–100 μL) to maintain concentration. Right panel: identical mass gradient with fixed medium volume (100 μL). (**B**,**C**) Corresponding fluorescence images for Lipofectamine 3000 (**B**) and jetPEI (**C**) under identical experimental configurations. (**D**–**F**) Quantitative analysis of eGFP expression intensity via ImageJ for HPAE (**D**), Lipofectamine 3000 (**E**), and jetPEI (**F**). (**G**–**I**) Cell viability was assessed by AlamarBlue assay post-transfection for HPAE (**G**), Lipofectamine 3000 (**H**), and jetPEI (**I**). Data are presented as mean ± SEM. *n* = 3 biological independent replicates. *p* values were determined via two-way ANOVA with Dunnett’s multiple comparisons test. ns: *p* > 0.05, * *p* < 0.05, **** *p* < 0.0001.

To systematically evaluate the regulatory role of plasmid DNA (pDNA) concentration in HPAE-mediated transfection, two experimental strategies were implemented in HEK293T cells. The first strategy employed a pDNA concentration gradient at a fixed culture volume (100 μL), while the second strategy used volume–concentration co-regulation, in which plasmid mass per well was held constant and concentration was increased by reducing the culture volume. Transfection efficiency and cytotoxicity were compared with Lipofectamine 3000 and jetPEI using an optimized polymer-to-DNA mass ratio of 20:1 (*w*/*w*). In the low plasmid mass range (0.2–0.5 μg/well), HPAE exhibited clear concentration-dependent transfection enhancement under both strategies. Quantitative analysis of eGFP expression (Figure 2A,D) showed that increasing the final concentration from 2 μg/mL to 5 μg/mL at a constant plasmid mass (0.2 μg/well) resulted in an approximately 33-fold increase in fluorescence intensity. At the same final concentration (5 μg/mL), transfection efficiency remained consistent across different plasmid mass groups, indicating that delivery performance was governed primarily by concentration rather than total DNA input. Flow cytometry analysis further confirmed this observation, showing comparable percentages of eGFP-positive cells across groups at the same concentration (Figure S10). Cell viability assays (Figure 2G) showed negligible cytotoxicity across this range, which was further supported by luciferase reporter assays (Figure S2A). Notably, the luciferase expression levels were consistent with the eGFP transfection results, further validating the concentration-dependent transfection behavior of HPAE. In stark contrast, Lipofectamine 3000 and jetPEI failed to achieve effective improvement in transfection efficiency when the medium volume was reduced to increase concentration.

**Figure 3:**
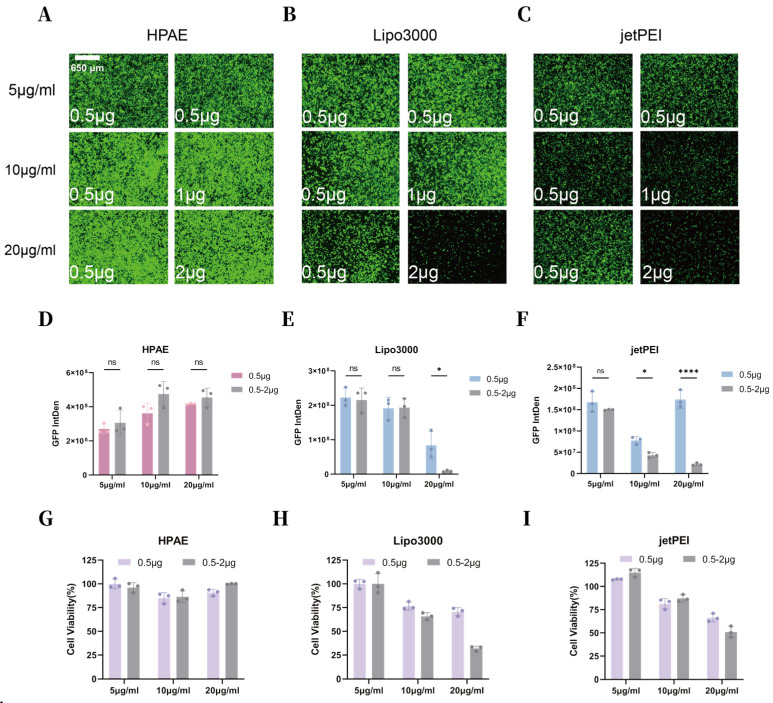
Enhanced transfection through concentration elevation with constant plasmid mass. (**A**) Fluorescence images of HEK293T cells transfected with HPAE/pDNA polyplexes. Left panel: constant plasmid mass (0.5–2 μg/well) with decreasing medium volume (100–25 μL) to achieve concentration multiplication. Right panel: identical mass gradient with fixed medium volume (100 μL). (**B**,**C**) Corresponding fluorescence images for Lipofectamine 3000 (**B**) and jetPEI (**C**) under identical configurations. (**D**–**F**) Quantitative analysis of eGFP expression intensity via ImageJ for HPAE (**D**), Lipofectamine 3000 (**E**), and jetPEI (**F**). (**G**–**I**) Cell viability assessment was done using an AlamarBlue assay for HPAE (**G**), Lipofectamine 3000 (**H**), and jetPEI (**I**). scale bar: 650 μm. Data are presented as mean ± SEM. *n* = 3 biological independent replicates. *p* values were determined via two-way ANOVA with Dunnett’s multiple comparisons test. ns: *p* > 0.05, * *p* < 0.05, **** *p* < 0.0001.

In the high plasmid mass range (0.5–2.0 μg/well), HPAE maintained robust transfection performance as concentration increased to 10 and 20 μg/mL via volume compression, whilst the plasmid mass remained fixed at 0.5 μg (Figure 3A,D). ImageJ-based fluorescence quantification revealed sustained concentration-dependent enhancement, with consistent transfection efficiency observed across different plasmid mass groups at the same final concentration. Flow cytometry analysis further confirmed these results, showing stable median fluorescence intensity and comparable percentages of eGFP-positive cells across different plasmid mass groups at the same concentration (Figure S11). Luciferase assays (Figure S2B) corroborated these findings. Cell viability remained high at concentrations up to 20 μg/mL, with only mild cytotoxic effects observed (Figure 3G). These results proved that HPAE enables efficient transfection while substantially mitigating cellular stress. In contrast, both Lipofectamine 3000 and jetPEI exhibited pronounced cytotoxicity and declining transfection efficiency at concentrations ≥ 10 μg/mL (Figure 3B,C,E,F,H,I). This indicates their unstable biocompatibility and unreliable safety under high-concentration transfection conditions [14,38]. These results show that HPAE supports efficient and concentration-tolerant transfection across a broad range of plasmid concentrations while maintaining favorable biocompatibility. This performance is closely associated with its previously observed stable particle size distribution, low sedimentation rate, and good suspension stability [37,39]. These physicochemical characteristics may contribute to sustaining continuous and uniform cell–plasmid interactions under varying concentration conditions [40], thereby providing support for concentration-regulated transfection behavior.

These results indicate that by reducing the culture medium volume to increase plasmid concentration, HPAE enables efficient delivery within a range of concentrations while minimizing resource consumption. In stark contrast, Lipofectamine 3000 and jetPEI show reduced efficiency and increased toxicity under the same conditions. It is important to note that Lipofectamine 3000 and jetPEI were used under their standard manufacturer-recommended conditions without re-optimization for concentration-dependent variations. The primary objective of this study is to investigate the intrinsic concentration-dependent delivery behavior of HPAE rather than to perform a fully optimized comparison among different transfection systems. Under these standardized conditions, HPAE consistently demonstrates robust performance across varying concentration regimes, suggesting that its observed advantages arise from its intrinsic physicochemical properties rather than experimental bias. While further optimization of commercial reagents may improve their performance under specific conditions, such optimization falls beyond the scope of the present study. This may be because they lack biocompatibility at high concentrations [41,42]. It should be noted that this study did not directly quantitatively detect plasmid DNA bound to the cell surface; instead, conclusions regarding the concentration effect were supported by indirect but consistent evidence, including the controlled regulation of culture medium volume. Although reducing the culture medium volume may introduce secondary factors such as changes in metabolite concentration or altered osmotic pressure conditions, the consistently high cell viability maintained in this study indicates that these effects did not play a dominant role [43,44]. Conversely, the significant correlation between plasmid concentration and transfection efficiency suggests that the elevation of local DNA concentration on the cell surface is the primary driving factor. Furthermore, even at high concentrations, free plasmid DNA alone generally fails to produce detectable transfection effects due to poor cell membrane permeability and the lack of carrier-mediated protection [45], highlighting the crucial role of HPAE in facilitating cellular uptake and gene expression. The strong agreement between eGFP and luciferase dual-reporter systems further confirms that HPAE maintains reliable concentration-dependent performance and low toxicity across the entire tested concentration range, overcoming the limitations of conventional transfection reagents. This feature makes HPAE particularly suitable for applications that demand high effective plasmid concentration, such as lentivirus production.

**Figure 4:**
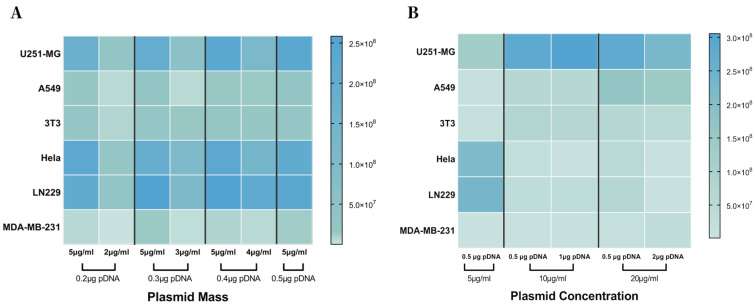
HPAE-mediated transfection efficiency across multiple cell lines under distinct plasmid delivery strategies. Quantitative fluorescence analysis by ImageJ of HPAE-mediated transfection across numerous cell types. (**A**) Fluorescence quantification by ImageJ under constant plasmid mass (0.2–0.5 μg) with increasing concentration (2–5 μg/mL). (**B**) Transfection performance with increasing plasmid mass (0.5–2 μg) at constant concentration (5–20 μg/mL). Tested cell lines: U251-MG (Human astrocytoma cells), A549 (Human lung adenocarcinoma cells), 3T3 (mouse fibroblast), HeLa (Human cervical cancer cells), LN229 (Human glioblastoma cells), and MDA-MB-231 (Human breast cancer cells). *n* = 3 biological independent replicates.

To assess whether the concentration-dependent transfection behavior of HPAE is conserved across different cell types, additional experiments were performed using eGFP-encoding plasmid DNA in a panel of cell lines (Figure 4). The tested cell lines included cancer cell lines (U251-MG, human glioma; A549, human non-small cell lung cancer cell; HeLa, human cervical cancer cell; LN229 human glioblastoma cell; MDA-MB-231, human breast cancer cell) and fibroblasts (3T3, mouse fibroblasts cell). Quantitative analysis of fluorescence images using ImageJ showed that eGFP expression increased by 2.4- to 38.9-fold as plasmid concentration increased (Figures S3–S8). Additionally, although lower transfection efficiency was observed in A549, MDA-MB-231, and 3T3 cells, these cell types still responded significantly to changes in plasmid concentration. This may be attributed to differences in adhesion properties, carrier compatibility, and endocytic pathways across cell types [46,47,48]. Cancer cells with high transfection efficiency typically exhibit elevated endocytic activity [49,50], while normal cells, such as fibroblasts, demonstrate lower internalization efficiency. The performance of the HPAE delivery system across multiple cell types shows that, although baseline transfection efficiency varies among cells, all exhibit a significant increase with rising plasmid concentration. This suggests that the concentration-dependent mechanism is not cell-type-specific but is a general principle of HPAE-mediated delivery.

### 3.3. Plasmid Concentration Modulates Uptake and Endosomal Escape of HPAE Polyplexes

**Figure 5:**
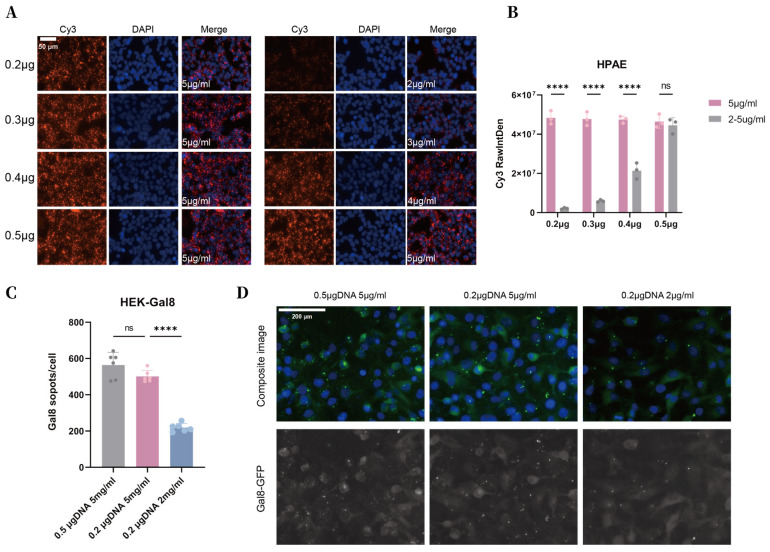
Effects of plasmid concentration on cellular uptake and endosomal escape efficiency mediated by HPAE/pDNA polyplexes. (**A**) Fluorescence images of HEK293T cells transfected with Cy3-labeled pDNA (red) using optimized transfection systems with indicated plasmid concentrations (scale bar: 50 μm). (**B**) Quantitative analysis of Cy3 fluorescence intensity by ImageJ (*n* = 3 biological independent replicates). (**C**) Quantifying Gal8 spots fluorescence per cell in HEK-Gal8 cells by ImageJ, reflecting endosomal membrane disruption efficiency under different plasmid concentrations (0.2–0.5 μg, 2–5 μg/mL) (*n* = 6 biological independent replicates). (**D**) Representative fluorescence images of Gal8 puncta (green) in HEK-Gal8 cells, showing increased endosomal escape at higher concentrations (scale bar: 200 μm). Data are presented as mean ± SEM. *p* values were determined via two-way (**B**) and one-way (**C**) ANOVA with Dunnett’s multiple comparisons test. ns: *p* > 0.05, **** *p* < 0.0001.

To characterize the effect of plasmid concentration on the cellular uptake of HPAE/pDNA polyplexes, we fluorescently labeled pDNA with Cy3 and complexed it with HPAE before treating HEK293T cells. Intracellular fluorescence signals were assessed via microscopic imaging to evaluate the level of polyplex uptake. Microscopic imaging (Figure 5A) revealed a strong positive correlation between pDNA uptake and concentration. When the concentration increased from 2 μg/mL to 5 μg/mL, cellular uptake levels markedly increased. However, when the mass of plasmid added per well was raised from 0.2 μg to 0.5 μg while maintaining the concentration at 5 μg/mL, no significant difference in uptake was observed. ImageJ-based quantification (Figure 5B) indicated that, at the same pDNA mass, the high-concentration group (5 μg/mL) exhibited a 22.9-fold higher Cy3 fluorescence intensity on the cell membrane compared with the low-concentration group (2 μg/mL). Furthermore, when both the plasmid mass and concentration were increased simultaneously (from 0.2 μg at 2 μg/mL to 0.5 μg at 5 μg/mL), the fluorescence intensity increased by 21.1-fold. This confirms that the transfection efficiency depends on the cellular uptake mechanism of the complexes, rather than merely the total mass of plasmid [51]. This concentration-dependent effect is likely associated with the stability of the HPAE/pDNA polyplexes and their adsorption capacity to the cell membrane [52].

Subsequently, HEK293T-Gal8-YEP cells were utilized. Upon carrier-mediated endosome disruption, Gal8-YEP rapidly redistributes from a diffuse cytoplasmic localization to accumulate as bright punctate structures, enabling real-time, label-free quantification of endosomolytic activity. For the Gal8-YFP reporter assay to evaluate endosomal membrane disruption, three representative transfection conditions were optimized and selected based on the eGFP transfection results: (i) 0.5 μg pDNA at 5 μg/mL, (ii) 0.2 μg pDNA at 5 μg/mL, and (iii) 0.2 μg pDNA at 2 μg/mL. These three groups were designed to distinguish the effects of pDNA mass from plasmid concentration, thereby fully capturing the impact of concentration changes. The results (Figure 5C,D) showed that comparable proportions of Gal8-positive cells were observed between the 0.5 μg/well and 0.2 μg/well groups at the same plasmid concentration (5 μg/mL). In contrast, when the total pDNA mass was maintained at 0.2 μg/well, reducing the culture volume from 100 μL (2 μg/mL) to 40 μL (5 μg/mL) resulted in a 2.28-fold increase in the proportion of Gal8-positive cells. These results indicate that increasing plasmid concentration by reducing the volume of the culture medium while maintaining a constant plasmid mass, cellular endosomal escape activity is significantly enhanced. However, quantitatively distinguishing the relative contributions of cellular uptake and endosomal escape to the increased Gal8-GFP signal remains challenging. Taken together, increasing plasmid concentration by reducing medium volume without altering DNA amount significantly enhances both cellular uptake and endosomal disruption. These effects are consistent with the observed concentration-dependent enhancement in transfection efficiency and may at least partially explain this phenomenon. Cellular uptake and endosomal escape represent two critical and sequential barriers in transfection [17,53], both of which can be improved through modulation of plasmid concentration. This coordinated enhancement highlights plasmid concentration as an effective and tunable parameter for overcoming key intracellular barriers in nonviral gene delivery.

### 3.4. Enhanced HPAE Transfection System for Large-Scale Lentivirus Production

**Figure 6:**
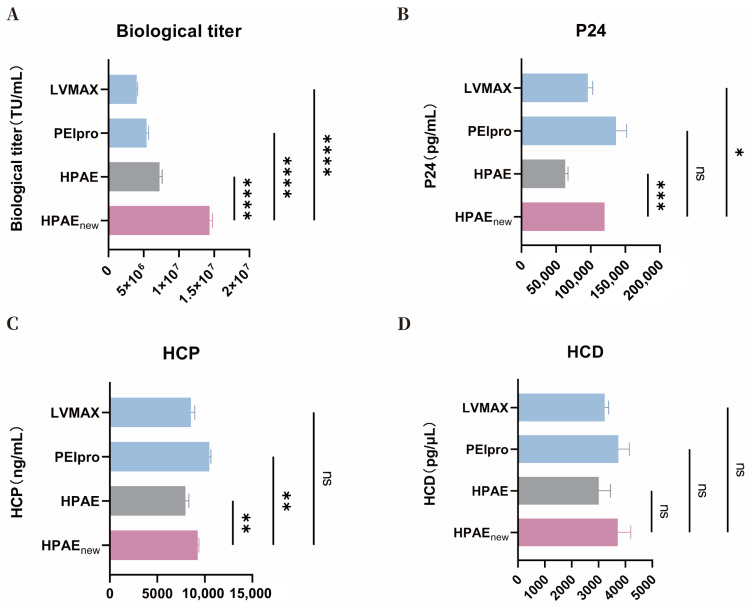
Comparison of lentivirus production and product quality across different transfection strategies. HEK293T cells were transfected with PEIpro, LVMAX, HPAE conventional strategy (HPAE), and HPAE new half-volume transfection–supplement strategy (HPAE_new_). (**A**) Functional viral titers were quantified by flow cytometry as transducing units (TU/mL). (**B**–**D**) Viral particle production and impurities were further evaluated by quantifying p24 levels (pg/mL) (**B**), host cell protein (HCP, ng/mL) (**C**), and host cell DNA (HCD, pg/μL) (**D**). Data are shown as mean ± SEM (*n* = 3 independent replicates). *p* values were determined via one-way ANOVA with Dunnett’s multiple comparisons test. ns: *p* > 0.05, * *p* < 0.05, ** *p* < 0.01, *** *p* < 0.001, **** *p* < 0.0001.

In gene and cell therapies, transfection technology is critical throughout the therapeutic production. The production of vectors such as AAV and LVV highly depends on the transfection efficiency of viral production cell lines like HEK293T [54,55]. Concentration-dependent intracellular delivery behavior provides a key theoretical basis for developing efficient viral production processes. Based on the aforementioned concentration regulation mechanism, we developed a novel half-volume transfection–supplement strategy (HPAE_new_): 1. Transfection stage: Use 50% of the conventional culture medium volume (7.5 mL) to form high plasmid concentration complexes. 2. Supplement stage: After 6 h of transfection, add an equal volume of fresh medium (final volume: 15 mL) to maintain cell viability. Control groups included conventional HPAE (15 mL system), PEIpro (Polyplus), and LVMAX (Thermo Fisher) commercial reagents (all operated according to standard protocols), covering both commonly used laboratory and industrial mainstream methods. The results (Figure 6A) showed that the HPAE_new_ group achieved a titer of 1.43 × 10^7^ TU/mL, which is 1.9-fold higher than that of conventional HPAE. Moreover, it outperformed commercial reagents, with titers 2.7-fold that of the PEIpro group and 3.6-fold that of the LVMAX group. Additionally, we performed comparative analysis using BrPERfect for Virus reagent, which yielded comparable viral titers to HPAE_new_, and both were significantly higher than those of the PEIpro and LVMAX systems (Figure S9). Importantly, the superior performance of HPAE_new_ underscores that high plasmid concentration during the critical transfection window is essential for maximizing lentivirus packaging efficiency, highlighting concentration tuning as a key determinant of productive viral output.

Furthermore, to evaluate the application potential of HPAE in lentiviral production, we compared p24 levels together with host cell protein (HCP) and host cell DNA (HCD) contents in the viral supernatant, enabling assessment of both production performance and impurity burden (Figure 6B–D). The results revealed significant differences in p24 levels among the strategies (Figure 6B). The HPAE_new_ group yielded the highest p24 concentration, significantly higher than conventional HPAE and LVMAX, and comparable to PEI. Regarding HCP content (Figure 6C), although HPAE_new_ exhibited marginally higher levels than conventional HPAE, it was comparable to that of LVMAX and remained significantly lower than PEIpro. It indicates acceptable impurity control while maintaining high production performance. For HCD residue (Figure 6D), no significant differences were observed between HPAE_new_ and the conventional HPAE, PEIpro, or LVMAX groups, indicating comparable performance in DNA residue control. The HPAE_new_ system achieves a good balance between increasing lentivirus yield and controlling impurity level, demonstrating its potential for large-scale lentivirus production. This strategy uses a simple yet effective method, adjusting the culture medium volume to regulate plasmid concentration, and it easily integrates into existing transfection and production processes. Also, this system provides a viable path to higher yields with the same resources and potentially lower production costs.

## 4. Conclusions

This study shows that plasmid concentration at the cell surface is a key regulator of transfection efficiency, independent of plasmid mass. Efficient gene delivery can be achieved by increasing plasmid concentration rather than plasmid amount, a behavior that is supported by the favorable physicochemical properties of the HPAE delivery system. This concentration-dependent effect is consistently observed across low and high dose conditions and is validated across multiple cell types, indicating that it is not cell-type specific. This is supported by evidence from cellular uptake and endosomal escape analyses. Applying this principle to lentiviral production, we developed a half-volume transfection–supplement strategy (HPAE_new_), the subsequent medium supplementation step alleviates concentration-associated cellular stress, enabling efficient plasmid uptake without compromising viability. This strategy provides a practical approach for more efficient lentivirus production by enhancing yield without compromising purity, while also reducing production costs.

## 5. Patents

The hyperbranched cationic polymer (HPAE) system used in this study and its application in nucleic acid delivery are related to previously disclosed patented technologies. Specifically, the design and development of the polymer materials are based in part on methodologies described in the following patent applications: hyperbranched cationic polymers for nucleic acid delivery (WIPO Patent Application WO 2021/058491 A1) and nanoparticle compositions for gene therapy (WIPO Patent Application WO 2021/058492 A1).

These patents primarily cover the structural design of polymeric carriers and their general application in gene delivery systems. The present study builds upon this foundation to further investigate the concentration-dependent delivery mechanism of HPAE and its application in lentiviral vector production. The experimental design, data analysis, and conclusions reported here are focused on addressing specific scientific questions and do not constitute a direct reproduction of the patented content.

## Data Availability

The original contributions presented in this study are included in the article and Supplementary Material. Further inquiries can be directed to the corresponding author.

## Supplementary Materials

The following supporting information can be downloaded at: https://www.mdpi.com/article/10.3390/polym18091015/s1, Figure S1: Optimization of heparin displacement conditions for different transfection reagents; Figure S2: Evaluation of concentration-dependent transfection efficiency using luciferase plasmids; Figure S3: Validation of concentration-dependent transfection behavior of HPAE in U251-MG cells; Figure S4: A549 cells; Figure S5: 3T3 cells; Figure S6: Hela cells; Figure S7: LN229 cells; Figure S8: MDA-MB-231 cells; Figure S9: Comparison of lentivirus production efficiency among BrPERfect for Virus and different transfection strategies. Figure S10: Flow cytometry analysis of transfection performance under the reduced plasmid mass with maintained concentration strategy. Figure S11: Enhanced transfection through concentration elevation at constant plasmid mass.
