# Supplementary material for: Concentration of DNA at the Cell Surface Dictates Transfection Efficacy: A Hyperbranched Poly(β-Amino Ester) Mediated Strategy for Enhanced Lentivirus Production
Source: Polymers (Basel). 2026 Apr 22;18(9):1015. doi: 10.3390/polym18091015 (PMC13165123; doi:10.3390/polym18091015)
Supplement: Supplementary file 1 [file polymers-18-01015-s001.zip › polymers-4230280-supplementary.pdf]

Supporting Information

Concentration of DNA at The Cell Surface Dictates Transfection Efficacy: A Hyperbranched Poly(β-amino ester) Mediated Strategy for Enhanced Lentivirus Production

Miao Wei[a,b] ‡, Liang Yao[g] ‡, Xinyue Wang[a,b], Meilin Guo[d], Haonan Li[e], Guang Chen[f], Xianqing Wang[g], Xi Wang[b,c,g], Wenxin Wang[b,c,g], Zhonglei He[b,c,f*]

‡These authors contributed equally in this work;

a School of Medicine, Anhui University of Science and Technology, Huainan 232001, China;

b Institute of Precision Medicine (AUST-IPM), Anhui University of Science and Technology, Huainan 232001, China;

c School of Public Health, Anhui University of Science and Technology, Huainan 232001, China;

d ST PHI Therapeutics Co., Ltd, Hangzhou Zhejiang 310051, China

e ACROBiosystems Inc., Beijing 100176, China;

f Zhejiang Key Laboratory of New Drug Development for Central Nervous System Diseases, School of Medicine, Taizhou University, Zhejiang 318000, P. R. China;

g Charles Institute of Dermatology, University College Dublin, Dublin 4, Ireland.

*Correspondence: Zhonglei He Email: hzlph@aust.edu.cn;

Supplementary figures

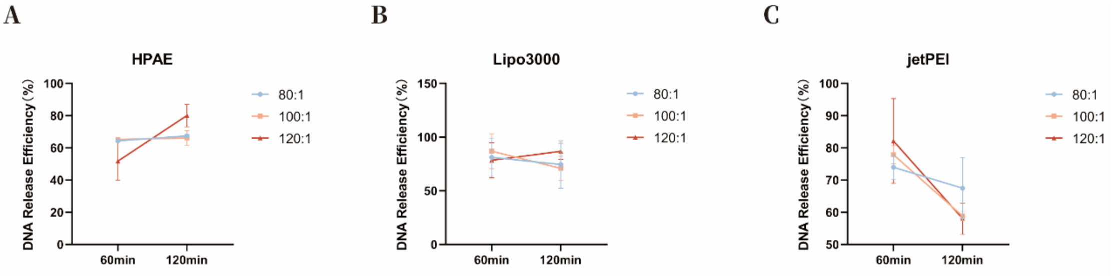

Fig. S1 Optimization of heparin displacement conditions for different transfection reagents.(A) HPAE/pDNA, (B) Lipofectamine 3000/pDNA, and (C) jetPEI/pDNA complexes were incubated with heparin at various heparin-to-DNA mass ratios (80:1, 100:1, 120:1) for 1 h and 2 h at 37 °C to determine the optimal conditions for complete DNA release(n=3 biological independent replicates). PicoGreen fluorescence assay was employed to quantify the dissociated DNA, with fluorescence intensity corresponding to the fraction of DNA released from the complexes. The conditions yielding maximal fluorescence were selected for subsequent sedimentation-stability experiments. Data are presented as mean ± SEM.

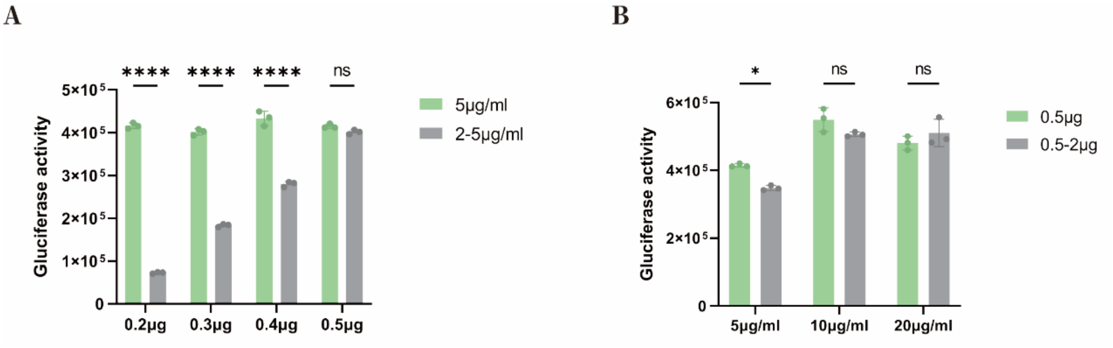

Fig. S2 Evaluation of concentration-dependent transfection efficiency using luciferase plasmids. HEK293T cells were transfected with luciferase-encoding plasmids, and luminescence intensity was measured 48 h post-transfection using a Firefly Luciferase Reporter Assay. (A) Transfection efficiency of HPAE/pDNA complexes at low plasmid input (0.2–0.5 μg per well) under concentrations (2–5 μg/mL). (B) Transfection efficiency at higher plasmid input (0.5–2 μg per well) under equivalent concentration gradients (5–20 μg/mL). Data are presented as mean ± SEM. n=3 biological independent replicates. P values were determined via two-way ANOVA with Dunnett's multiple comparisons test. ns: P > 0.05, *P < 0.05, **P < 0.01, ***P < 0.001, ****P < 0.0001.

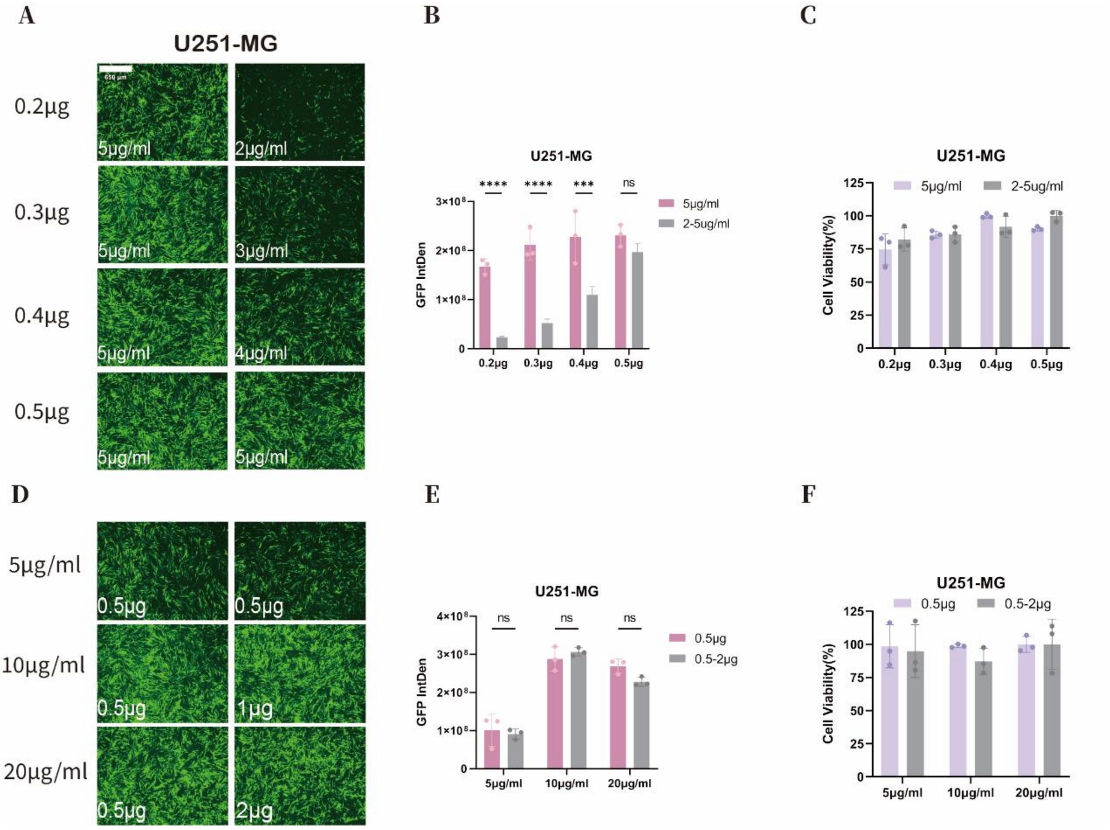

Fig. S3 Validation of concentration-dependent transfection behavior of HPAE in U251-MG cells. (A, D) Representative fluorescence microscopy images showing eGFP expression after transfection with

HPAE/pDNA complexes at different plasmid input levels. Panels (A–C) correspond to the low-DNA range (0.2–0.5 μg per well, 2–5 μg/mL), while panels (D–F) represent the high-DNA range (0.5–2 μg per well, 5–20 μg/mL). (B, E) Quantitative analysis of eGFP fluorescence intensity was performed using ImageJ software.(C, F) Cell viability was determined 48 h post-transfection by AlamarBlue assay.Data are presented as mean ± SEM. n=3 biological independent replicates. P values were determined via two-way ANOVA with Dunnett’s multiple comparisons test. ns: P > 0.05, *P < 0.05, **P < 0.01, ***P < 0.001, ****P < 0.0001.

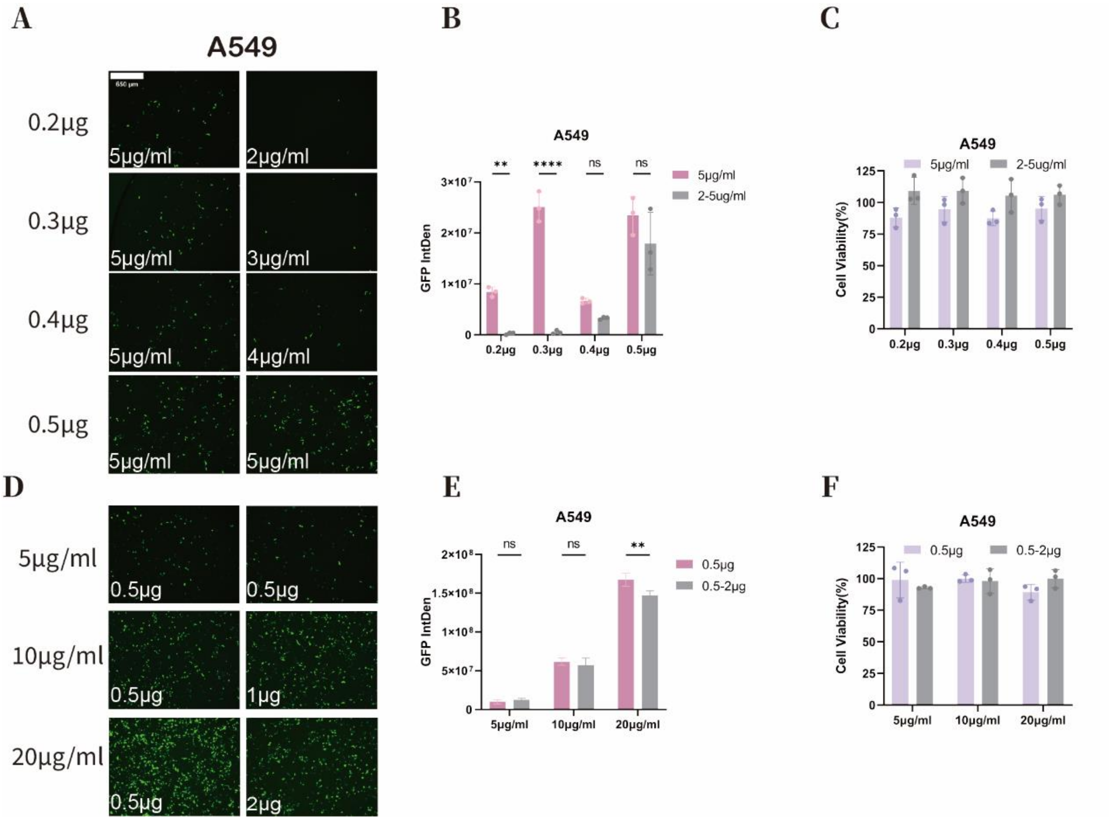

Fig. S4 Validation of concentration-dependent transfection behavior of HPAE in A549 cells. (A, D) Representative fluorescence microscopy images showing eGFP expression after transfection with HPAE/pDNA complexes at different plasmid input levels. Panels (A–C) correspond to the low-DNA range (0.2–0.5 μg per well, 2–5 μg/mL), while panels (D–F) represent the high-DNA range (0.5–2 μg per well, 5–20 μg/mL). (B, E) Quantitative analysis of eGFP fluorescence intensity was performed using ImageJ software. (C, F) Cell viability was determined 48 h post-transfection by AlamarBlue assay. Data are presented as mean ± SEM. n=3 biological independent replicates. P values were determined via two-way ANOVA with Dunnett’s multiple comparisons test. ns: P > 0.05, *P < 0.05, **P < 0.01, ***P < 0.001, ****P < 0.0001.

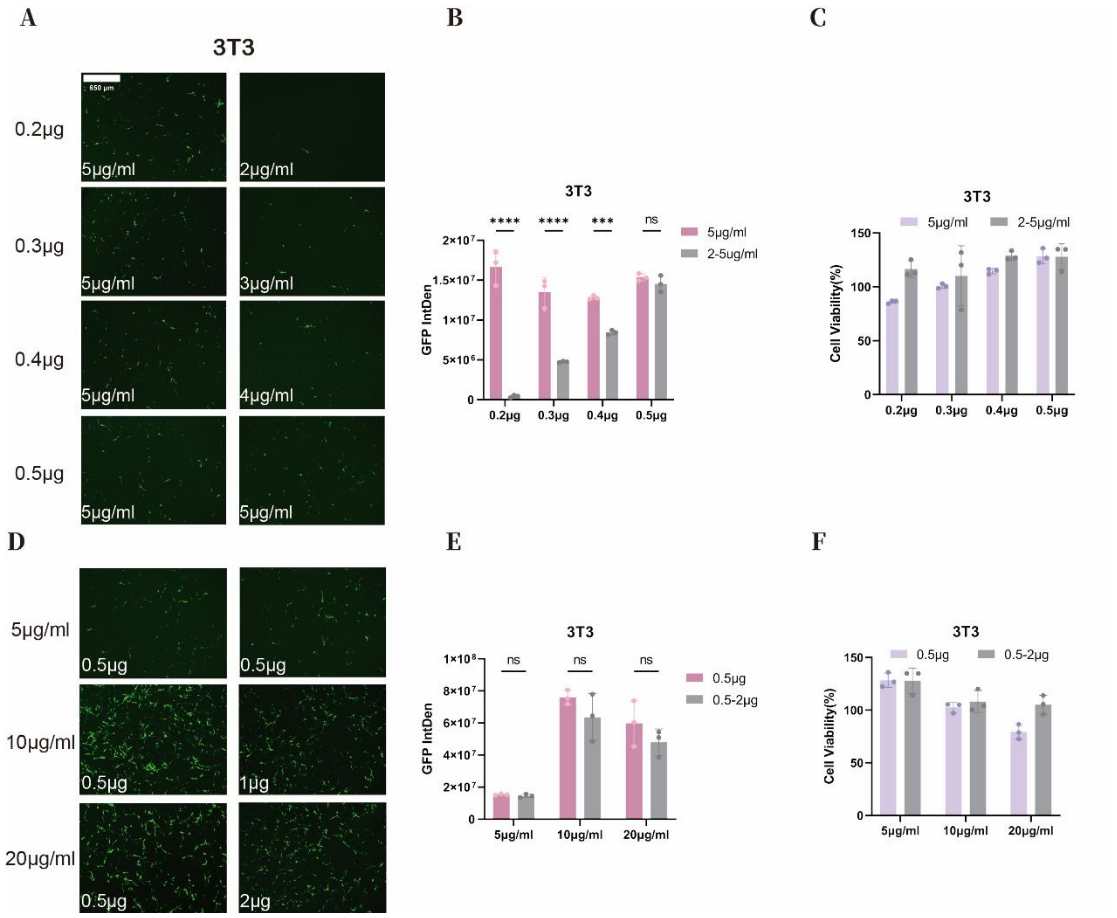

Fig. S5 Validation of concentration-dependent transfection behavior of HPAE in 3T3 cells. (A, D) Representative fluorescence microscopy images showing eGFP expression after transfection with HPAE/pDNA complexes at different plasmid input levels. Panels (A–C) correspond to the low-DNA range (0.2–0.5 μg per well, 2–5 μg/mL), while panels (D–F) represent the high-DNA range (0.5–2 μg per well, 5–20 μg/mL). (B, E) Quantitative analysis of eGFP fluorescence intensity was performed using ImageJ software. (C, F) Cell viability was determined 48 h post-transfection by AlamarBlue assay. Data are presented as mean ± SEM. n=3 biological independent replicates. P values were determined via two-way ANOVA with Dunnett's multiple comparisons test. ns: $P > 0.05$, $^*P < 0.05$, $^{**}P < 0.01$, $^{***}P < 0.001$, $^{****}P < 0.0001$.

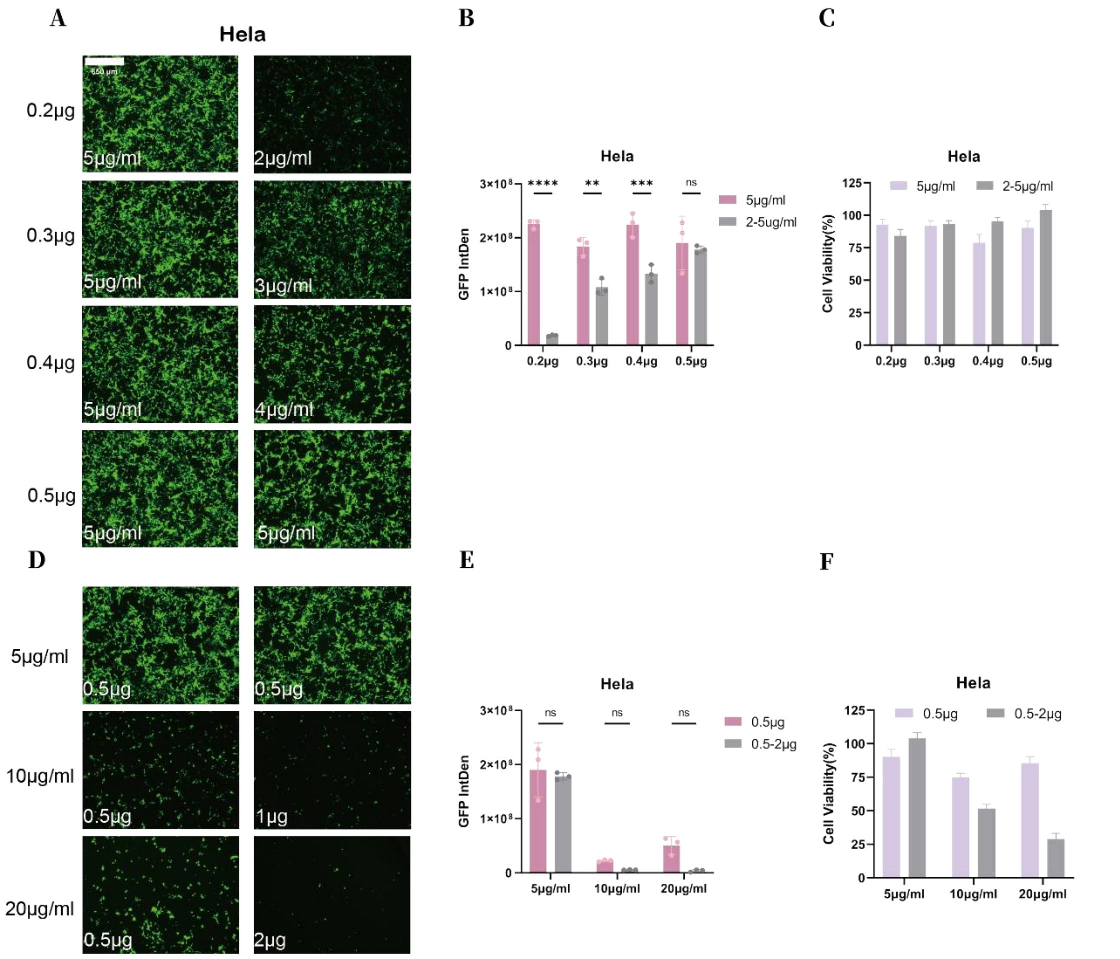

Fig. S6 Validation of concentration-dependent transfection behavior of HPAE in Hela cells. (A, D) Representative fluorescence microscopy images showing eGFP expression after transfection with HPAE/pDNA complexes at different plasmid input levels. Panels (A–C) correspond to the low-DNA range (0.2–0.5 μg per well, 2–5 μg/mL), while panels (D–F) represent the high-DNA range (0.5–2 μg per well, 5–20 μg/mL). (B, E) Quantitative analysis of eGFP fluorescence intensity was performed using ImageJ software. (C, F) Cell viability was determined 48 h post-transfection by AlamarBlue assay. Data are presented as mean ± SEM. n=3 biological independent replicates. P values were determined via two-way ANOVA with Dunnett's multiple comparisons test. ns: $P > 0.05$, $^{*}P < 0.05$, $^{**}P < 0.01$, $^{***}P < 0.001$, $^{****}P < 0.0001$.

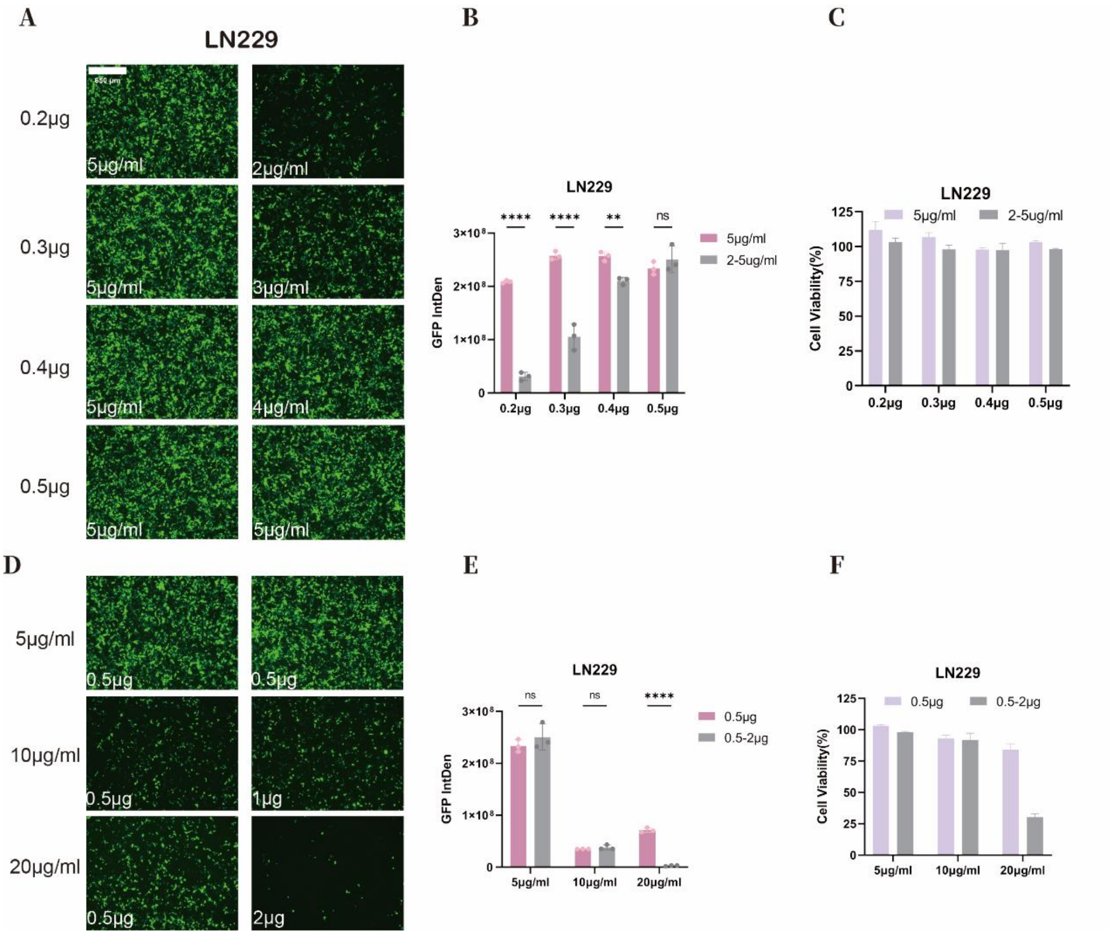

Fig. S7 Validation of concentration-dependent transfection behavior of HPAE in LN229 cells. (A, D) Representative fluorescence microscopy images showing eGFP expression after transfection with HPAE/pDNA complexes at different plasmid input levels. Panels (A–C) correspond to the low-DNA range (0.2–0.5 μg per well, 2–5 μg/mL), while panels (D–F) represent the high-DNA range (0.5–2 μg per well, 5–20 μg/mL). (B, E) Quantitative analysis of eGFP fluorescence intensity was performed using ImageJ software. (C, F) Cell viability was determined 48 h post-transfection by AlamarBlue assay. Data are presented as mean ± SEM. n=3 biological independent replicates. P values were determined via two-way ANOVA with Dunnett's multiple comparisons test. ns: $P > 0.05$, $^{*}P < 0.05$, $^{**}P < 0.01$, $^{***}P < 0.001$, $^{****}P < 0.0001$.

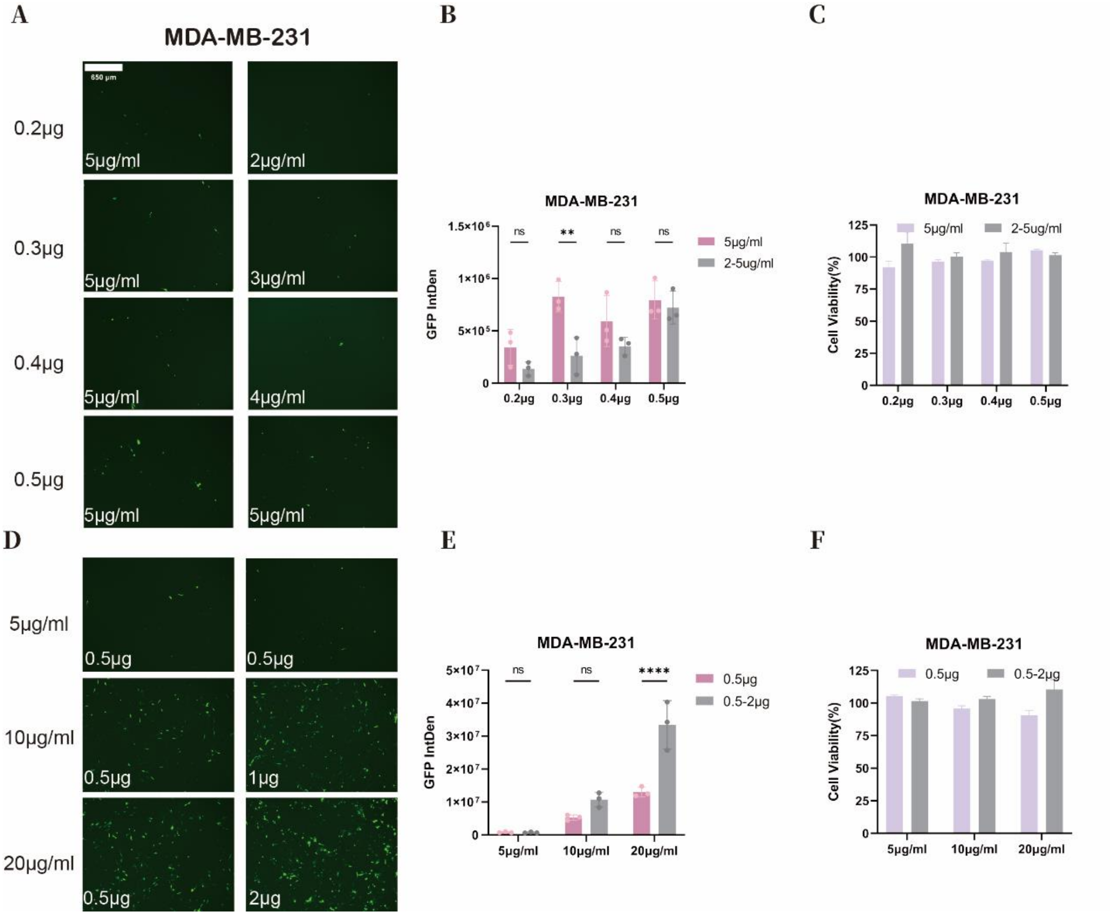

Fig. S8 Validation of concentration-dependent transfection behaviour of HPAE in MDA-MB-231 cells. (A, D) Representative fluorescence microscopy images showing eGFP expression after transfection with HPAE/pDNA complexes at different plasmid input levels. Panels (A–C) correspond to the low-DNA range (0.2–0.5 μg per well, 2–5 μg/mL), while panels (D–F) represent the high-DNA range (0.5–2 μg per well, 5–20 μg/mL). (B, E) Quantitative analysis of eGFP fluorescence intensity was performed using ImageJ software. (C, F) Cell viability was determined 48 h post-transfection by AlamarBlue assay. Data are presented as mean ± SEM. n=3 biological independent replicates. P values were determined via two-way ANOVA with Dunnett's multiple comparisons test. ns: $P > 0.05$, $^{*}P < 0.05$, $^{**}P < 0.01$, $^{***}P < 0.001$, $^{****}P < 0.0001$.

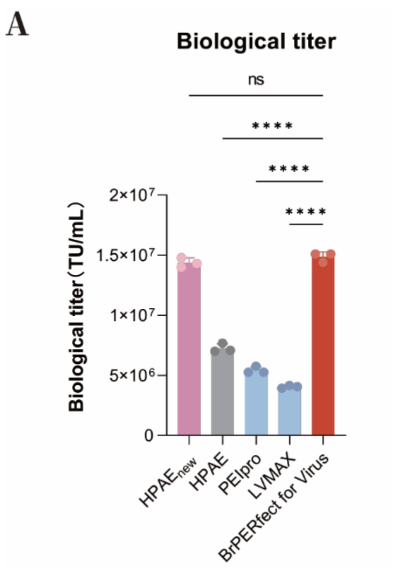

Fig. S9 Comparison of lentivirus production efficiency among BrPERfect for Virus and different transfection strategies. HEK293T cells were transfected using five transfection systems: BrPERfect for Virus, HPAEnew (half-volume transfection–supplement strategy), conventional HPAE, PEIpro, and LVMAX. (A) Functional viral titers were quantified by flow cytometry as transducing units (TU/mL). Data are presented as mean ± SEM. n=3 biological independent replicates. P values were determined via one-way ANOVA with Dunnett's multiple comparisons test. ns: P > 0.05, *P < 0.05, **P < 0.01, ***P < 0.001, ****P < 0.0001.

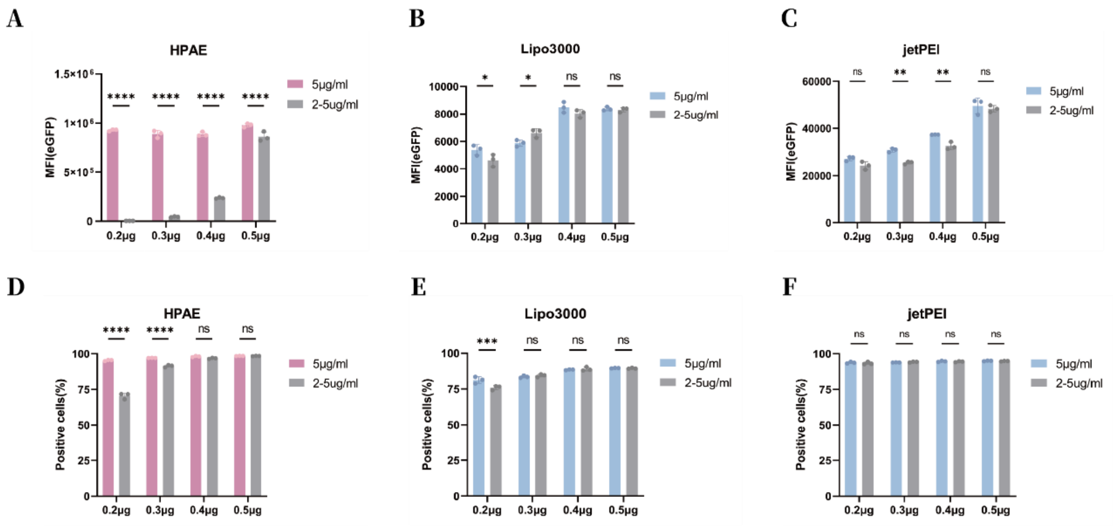

Fig. S10 Flow cytometry analysis of transfection performance under the reduced plasmid mass with maintained concentration strategy. (A–C) Median fluorescence intensity (MFI) of eGFP expression in cells transfected with HPAE (A), Lipofectamine 3000 (B), and jetPEI (C) under different plasmid mass conditions (0.2–0.5 μg) at two concentrations (5 μg/mL and 2–5 μg/mL). (D–F) Percentage of eGFP-positive cells corresponding to HPAE (D), Lipofectamine 3000 (E), and jetPEI (F)

under the same conditions. Data are presented as mean ± SEM (n = 3). Statistical significance was analyzed using two-way ANOVA with Dunnett’s multiple comparisons (ns, not significant; *$p < 0.05$; **$p < 0.01$; ***$p < 0.001$; ****$p < 0.0001$).

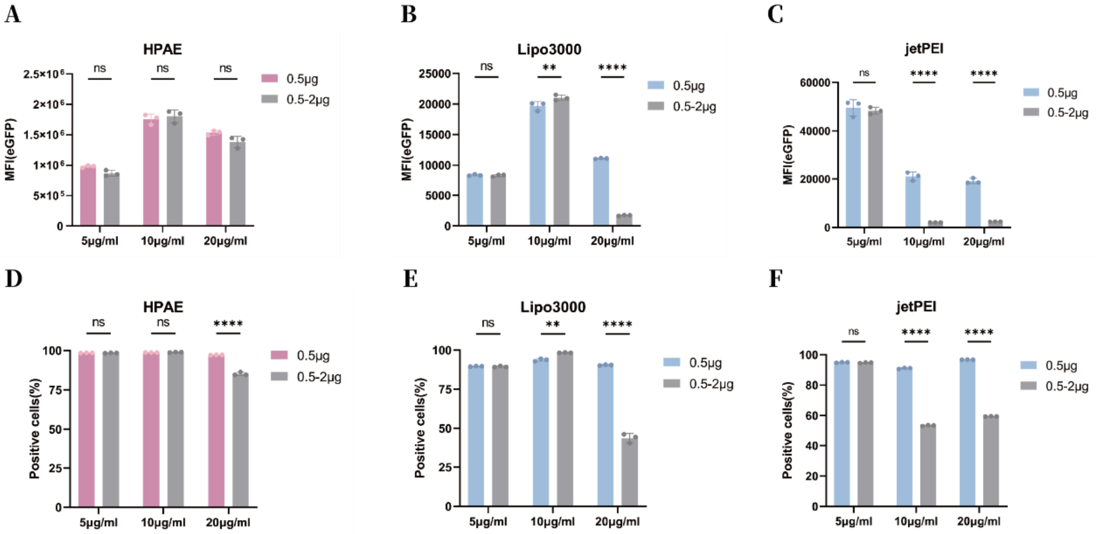

Fig. S11 Enhanced transfection through concentration elevation at constant plasmid mass. (A–C) Median fluorescence intensity (MFI) of eGFP expression in cells transfected with HPAE (A), Lipofectamine 3000 (B), and jetPEI (C) under increasing plasmid concentrations (5, 10, and 20 μg/mL) while maintaining a constant plasmid mass (0.5 μg). A comparison group (0.5–2 μg) is included to evaluate the effect of increased DNA input at corresponding concentrations. (D–F) Percentage of eGFP-positive cells for HPAE (D), Lipofectamine 3000 (E), and jetPEI (F) under the same conditions. Data are presented as mean ± SEM (n = 3). Statistical significance was analyzed using two-way ANOVA (ns, not significant; **$p < 0.01$; ****$p < 0.0001$).
